# Burden of VSD associated aortic valve cusp prolapse with aortic regurgitation and the impact of early surgery on clinical outcomes in South Asia

**DOI:** 10.12669/pjms.37.5.4845

**Published:** 2021

**Authors:** Sahrai Saeed, Yaso Emmanuel

**Affiliations:** 1 Sahrai Saeed, Department of Heart Disease, Haukeland University Hospital, Bergen, Norway; 2 Yaso Emmanuel, Cardiovascular Department, Guy’s and St Thomas’ Hospital, London, UK

Congenital heart disease (CHD) is defined as a structural defect in the heart or the great vessels present at birth. It includes a range of conditions, ventricular septal defect (VSD) being one of them (*Figure 1*), which is mostly diagnosed and when indicated, is treated in childhood.^1^,^2^ Undetected VSDs are associated with higher mortality and morbidity. VSDs may be either simple/isolated or complex and co-present with other cardiac lesions.^3^ VSDs can be divided into subgroups according to the location in the right ventricle: membranous (approximately 80% of VSDs - these can extend into the outlet, trabecular or inlet portions of the septum), muscular/trabecular (15-20%) and outlet sub-arterial defects.^1^,^2^ VSDs that involve the outlet septum may be associated with progressive aortic regurgitation (AR) due to prolapse of the right aortic cusp and aneurysm of the sinus of Valsalva.^4^ It has been shown that up to 40% patients with VSD-associated right aortic cusp prolapse can develop AR.^5^ A simple VSD may close spontaneously and in these patients survival rates are very high.^3^ VSD closure is indicated when there is associated haemodynamically significant left ventricular volume overload secondary to the shunt and in the absence of significant pulmonary hypertension.^1^,^2^ Progressive aortic cusp prolapse and regurgitation is a class IIa indication for VSD closure^1^,^2^ in order to try to preserve the aortic valve.

**Fig.1:**
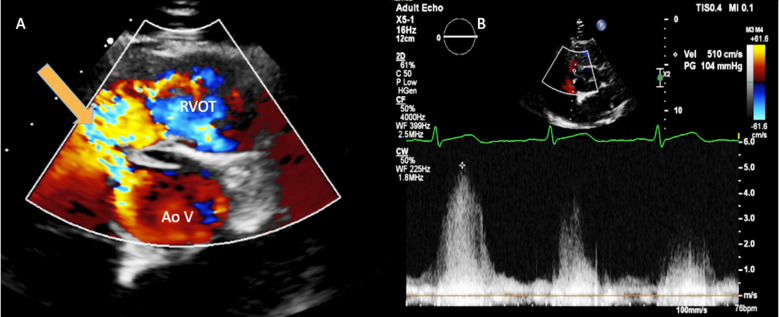
Panel A: Parasternal short axis view with colour Doppler showing the left to right jet through the VSD (red arrow). Panel B: Continuous wave Doppler trace on parasternal short axis view through the jet indicating high velocity left to right flow at 5.0 m/s consistent with a restrictive VSD. VSD, ventricular septal defect.

Patients with small defects that are not deemed to require closure in childhood should remain under follow-up as there can be sequelae, including progressive AR, progressive left ventricular dilatation, pulmonary hypertension, progressive muscle bundle formation in the right ventricular outflow tract leading to the development of a double-chambered right ventricle, and infective endocarditis may also occur as a result of jet lesions in the right ventricle^1^,^2^ Karonis *et al*. reported on the adult outcomes in a UK population with small VSDs that did not require closure in childhood who were under follow-up at a specialist centre.^6^ They identified 231 patients and 83% had defects involving either the perimembranous septum or sub-arterial septum, the remaining 17% had muscular defects. Overall long-term outcomes were good with no mortality and 97% were asymptomatic. However, 26 patients (11%) did require surgery for associated complications mostly for the development of a double chambered right ventricle (n=17, 65%) and infective endocarditis (n=6, 23%). Only two patients required surgery for progressive left to right shunting. AR was identified in 26 patients, (11%) and in these patients this was graded as mild in 21 and more than mild in the remaining five, but only one patient required surgery for progressive AR.

VSD with aortic cusp prolapse and subsequent AR is rare (approximately 5%) in North America and Western Europe, but is more common in the Asian populations.^4^,^5^,^7^,^8^ Kumari *et al*. reviewed a South Asian population with VSDs over a two year period and identified AR in 23% of patients.^9^ In this study, 17.9% of defects closed spontaneously, 51.6% of patients underwent surgery and in 30.5% of patients there was no indication for surgery. The prevalence of AR significantly reduced following surgery, however, the incidence of later AR in the un-operated patients is not known in this study.

Over the past two decades, the diagnostic imaging modalities and therapeutic approaches have improved, leading to better outcomes in CHD patients. In a prospective study of 7038 patients <16 years undergoing surgical treatment for CHD in Norway, cumulative survival until 16 years of age with complex CHD was 62.4% between 1971 to 1989 and 86.9% between 1990 to 2011 (p<0.0001). This trend was also particularly evident for patients with atrioventricular septal defects.^10^ However, the prevalence of VSD-associated aortic cusp prolapse and associated AR, and burden of residual AR following VSD surgery were not reported. Probably, this reflects the relatively low prevalence of VSD-associated AR in Scandinavian populations compared with south Asians. Internationally, given the overall continued improvement in surgical outcomes, some argue for earlier intervention on VSDs with aortic valve prolapse in order to prevent AR.

In a recent edition of the *Pakistan Journal of Medical Science (Pak J Med Sci)*, Waqar *et al*. presented some interesting results for surgical outcomes of repair of VSD-associated aortic valve prolapse.^4^ The authors studied 72 patients with VSD-associated aortic valve prolapse operated in Punjab Institute of Cardiology between 2016 and 2020 (18 patients per year). In 55 patients, AR was either absent (n=15, group I) or mild (n=40, group II). In 17 patients VSD was either associated with moderate AR (n=10, group III) or severe AR (n=7, group IV). VSD was closed in all patients, but AR was treated surgically in 17 patients with moderate or severe AR (group III-IV). After a mean follow-up of 3 to 3.5 years, the outcome were excellent with no late mortalities: in majority of patients, residual AR was either absent or not significant (trace or mild), while only in two patients AR was considered clinically significant and scheduled for redo operations. The authors in this study focused on patients with aortic valve involvement and the outcomes in groups I and II appeared good on the face of it in terms of the lack of AR at follow-up. However it is not clear what the indication for surgery was in the first place in these groups. Both of these groups underwent VSD closure only and did not require valve repair. If closure was indicated on the basis of a haemodynamically significant shunt, then this would not be deemed ‘early’ closure. Previous work,^11^ also published in the *Pakistan Journal of Medical Science* by the same author group but from a different tertiary heart center, reported on patients with sub-arterial VSD undergoing surgery. As with the most recent work, the study population was similarly classified according to the degree of aortic valve involvement. Similar results were reported over the short follow-up period (1.5-2.8 years). Again the indication for surgery in patients with no significant AR was not clear. Overall, the studies of Waqar *et al*.^4^,^11^ deserve appreciation and show that mid-term outcomes are excellent in CHD patients. This reflects their surgical skills and experiences at a “high-volume” tertiary center. However, it is important to note that in both studies, the indication for surgery in patients without significant or progressive AR was not clear. Without knowing more about the indication for surgery, it is difficult to address the question of whether there is a role for early isolated VSD closure before progressive regurgitation develops in order to preserve the valve and avoid valve repair, if closure is not indicated on haemodynamic grounds.

From previous European studies it is known that in surgically treated VSD patients, AR can occur late in follow-up and is a major subject of concern. Given the relatively short follow-up time in Waqar *et al*.’s most recent cohort, it is probably too early to evaluate occurrence of late AR. For comparison, results from a study in the Netherlands showed that the occurrence of AR after VSD surgery was worrying. Although overall survival was good (86% at 40 years), the percentage of patients with mild or moderate AR increased from 10% to 21% over the last 20 years of follow-up.^12^

The data from Karonis *et al*. 6 suggest that with small defects, the incidence of late aortic valve involvement is low. Additional longer follow-up studies in different populations to evaluate the late development of AR and other complications that require surgical intervention in patients in whom closure was not indicated in childhood will be helpful. The results reported by Waqar *et al*.^4^,^11^ appear promising, however, longer follow-up of patients with aortic valve involvement but only undergoing isolated VSD closure is required both to assess for late AR and to identify whether the incidence of other associated complications or need for surgical re-intervention is less than in patients with small defects who are un-operated. The data on longer term outcomes of both these groups are needed to determine the role of early surgical intervention to preserve the aortic valve.

## Authors Contribution

**SS** wrote the first draft of the article, which was subsequently revised for important scientific content by **YE**. Both authors approved the final submission.

## References

[1] Stout KK, Daniels CJ, Aboulhosn JA, Bozkurt B, Broberg CS, Colman JM (2019). 2018 AHA/ACC Guideline for the Management of Adults With Congenital Heart Disease:A Report of the American College of Cardiology/American Heart Association Task Force on Clinical Practice Guidelines. Circulation.

[2] Baumgartner H, De Backer J, Babu-Narayan SV, Budts W, Chessa M, Diller GP (2021). ESC Scientific Document Group. 2020 ESC Guidelines for the management of adult congenital heart disease. Eur Heart J.

[3] van der Bom T, Bouma BJ, Meijboom FJ, Zwinderman AH, Mulder BJ (2012). The prevalence of adult congenital heart disease, results from a systematic review and evidence based calculation. Am Heart J.

[4] Waqar T, Rizvi MFA, Nasir JA, Khan K (2021). Surgical outcome of repair of aortic valve prolapse and regurgitation associated with ventricular septal defect. Pak J Med Sci.

[5] Iwashima S, Uchiyama H, Ishikawa T, Takigiku K, Takahashi K, Toyono M (2017). Measurement of Aortic Valve Coaptation and Effective Height Using Echocardiography in Patients with Ventricular Septal Defects and Aortic Valve Prolapse. Pediatr Cardiol.

[6] Karonis T, Scognamiglio G, Babu-Narayan S, Uebing Diller G (2016). Clinical course and potential complications of small ventricular septal defects in adulthood:late development of left ventricular dysfunction justifies lifelong care. Int J Cardiol.

[7] Soto B, Becker AE, Moulaert AJ, Lie JT, Anderson RH (1980). Classification of ventricular septal defects. Br Heart J.

[8] Ando M, Takao A (1986). Pathological anatomy of ventricular septal defect associated with aortic valve prolapse and regurgitation. Heart Vessels.

[9] Kumari V, Shaikh AS, Zakai SB, Kumar N, Bangash SK, Patel N (2019). Incidence of Aortic Regurgitation in Association with Type of Ventricular Septal Defects and its Immediate and Intermediate Outcome after Surgical Closure. Cureus.

[10] Erikssen G, Liestøl K, Seem E, Birkeland S, Saatvedt KJ, Hoel TN (2015). Achievements in congenital heart defect surgery:a prospective, 40-year study of 7038 patients. Circulation.

[11] Waqar T, Rizvi MFA, Baig AR (2017). Doubly committed Subarterial Ventricular Septal defect repair:An experience of 51 cases. Pak J Med Sci.

[12] Menting ME, Cuypers JA, Opić P, Utens EM, Witsenburg M, van den Bosch AE (2015). The unnatural history of the ventricular septal defect:outcome up to 40 years after surgical closure. J Am Coll Cardiol.

